# Insights on bio-degumming of kenaf bast based on metagenomic and proteomics

**DOI:** 10.1186/s12864-020-6531-2

**Published:** 2020-02-03

**Authors:** Sheng Wen Duan, Li Feng Cheng, Xiang Yuan Feng, Qi Yang, Zhi Yuan Liu, Ke Zheng, Yuan De Peng

**Affiliations:** Institute of Bast Fiber Crops, Chinese Academy of Agriculture Sciences, Changsha, 410000 China

**Keywords:** Bio-degumming, *Hibiscus cannabinus*, Microbial diversity, iTRAQ

## Abstract

**Background:**

Microbes play important roles in kanef-degumming. This study aims at identifying the key candidate microbes and proteins responsible for the degumming of kenaf bast (*Hibiscus cannabinus*). Kenaf bast was cut into pieces and immersed into microbia fermentation liquid collected from different sites. Fermentation liquid samples were collected at 0, 40, 110 and 150 h and then subjected to the 16S/18S rRNA sequencing analysis and isobaric tag for relative and absolute quantitation (iTRAQ) analysis. The microbial (bacterial and fungal) diversity and the differentially expressed proteins/peptides (DEPs) were identified.

**Results:**

With the prolonged degumming time, the weight loss rate increased, the bacterial diversity was decreased. [Weeksellaceae], *Enterobacteriaceae* and *Moraxellaceae* were rapidly increased at 0~40 h, and then decreased and were gradually replaced by *Bacteroidaceae* from 40 h to 150 h. Similarly, *Chryseobacterium* and *Dysgonomonas* were gradually increased at 0~110 h and then decreased; *Acinetobacter* and *Lactococcus* were increased at 0~40 h, followed by decrease. *Bacteroides* was the dominant genus at 150 h. Sequencing 18S rRNA-seq showed the gradually decreased *Wallemia hederae* and increased *Codosiga hollandica* during degumming. iTRAQ data analysis showed Rds1, and pyruvate kinase I was decreased and increased in the kanef-degumming, respectively. Other DEPs of ferredoxin I, superoxide dismutase and aconitatehydratase were identified to be related to the Glyoxylate and dicarboxylate metabolism (ko00630).

**Conclusions:**

Bacteria including *Chryseobacterium*, *Dysgonomonas*, *Acinetobacter*, *Lactococcus* and *Bacteroides*and fungi like *Wallemia hederae* and *Codosiga hollandica* are key candidate microbes for kanef degumming.

## Background

Kenaf (*Hibiscus cannabinus*),which contains 8–16% lignin, 53–66% cellulose, 23–35% pectin and some hemicellulose, is an annual herbaceous bast fiber crop of the genus Malvaceae [1–3]. It is widely planted around the world, especially in the tropical and subtropical regions, such as Asia and Latin America. Kenaf fiber is widely used as an important basic raw material in textile, manufacturing and composite fabrication due to its strong pulling force [1, 4]. However, the retting methods can influence the quality of kenaf fiber.

Retting based on the intervention of bacteria and microbia enzymes promotes the development of the textile industry via resulting in a better quality of fibers. Conventional methods for the degumming of kenaf bast included traditional natural fermentation (water retting) and chemical degumming. In comparison with the natural fermentation and chemical degumming, biological (bacterial and enzymatic) degumming presents a series of advantages including high efficiency, low pollution, low cost and high fiber quality [3, 5–7]. The secretion of bacteria promote the decomposition of material, which can be used for bacteria to continue to grow [6, 8]. Ideal bacterial strains for kenaf degumming should have the advantages of secreting pectinase, hemicellulose, and ligninase, but not cellulase [6–8].

The screening of superior bacterial strains with the activity of pectate lyase, pectinase, hemicellulase and/or ligninase and the preservation of the natural fiber structure and mechanical properties is crucial for biological degumming [7–9]. A series of bacterial strains have been identified with strong ability of retting or degumming, like *Bacillus cereus* hn1–1 [10], *B. pumilus* [7], *B. licheniformis* and *B. subtilis* [11] and *B. tequilensis* SV11-UV37 [6]. Cheng et al. [10] showed that the 10 h-degumming process by *B. cereus* hn1–1 produced a residual gum rate as low as 5% and the fiber rate as high as 76%. Mao et al. [12] reported that the ramie retting could be completed within 56 h by using a microbia consortium RAMCD407 plus 0.2% NaOH, with 2.84% residual gum content and 5.2 cN/dtex breaking strength of the final fiber. In addition, our previous study [7] identified that pectinase and mannanase were the key enzymes in the degumming of kenaf bast mediated by bacteria including *B. pumilus, B. alcalophilus, Clostridium tertium, Brevibacillus brevis, Pectobacterium carotovora, Erwinia chrysanthemi, and Tyromyces subcaesius*. All these results suggested the pivotal roles of bacteria in the degumming of kenaf bast. However, there was no systematic analysis for the alterations of bacterial secretome during degumming of kenaf bast.

This study was performed to identify the key candidate microbes and secretory proteins during the retting and degumming of kenaf bast. Alterations of microbial proteomics and community during retting and degumming of kenaf bast was detected using isobaric tags for relative and absolute quantitation (iTRAQ) and 16S/18S rRNA sequencing, respectively. These findings provide novel insights into the retting and degumming of kenaf bast.

## Results

### Degumming of kenaf bast and bacteria collection

The weight loss rate of kenaf bast was gradually increased with degumming, ranging from 11.72% at 40 h and 32.06% at 190 h (Table 1). The bacterial viable count, however, was primarily decreased from initial 4.2 × 10^7^ CFU/ml to 8.7 × 10^6^ CFU/ml at 40 h post fermentation. It was increased to the maximum 5.1 × 10^8^ CFU/ml at 150 h, followed with a decrease. These results might suggest that the growth of bacteria had degumming function.

**Table 1:** Kenaf bast degumming effect during different enrichment time

Terms	0 h	40 h	110 h	150 h	190 h
Weight loss rate (%)	–	11.72	24.45	31.26	32.06
Initial content of live bacterial (CFU/mL)	4.2 × 10^7^	8.7 × 10^6^	7.2 × 10^7^	5.1 × 10^8^	3.1 × 10^8^

### General characteristics of 16S/18S rRNA sequencing

**Fig. 1:**
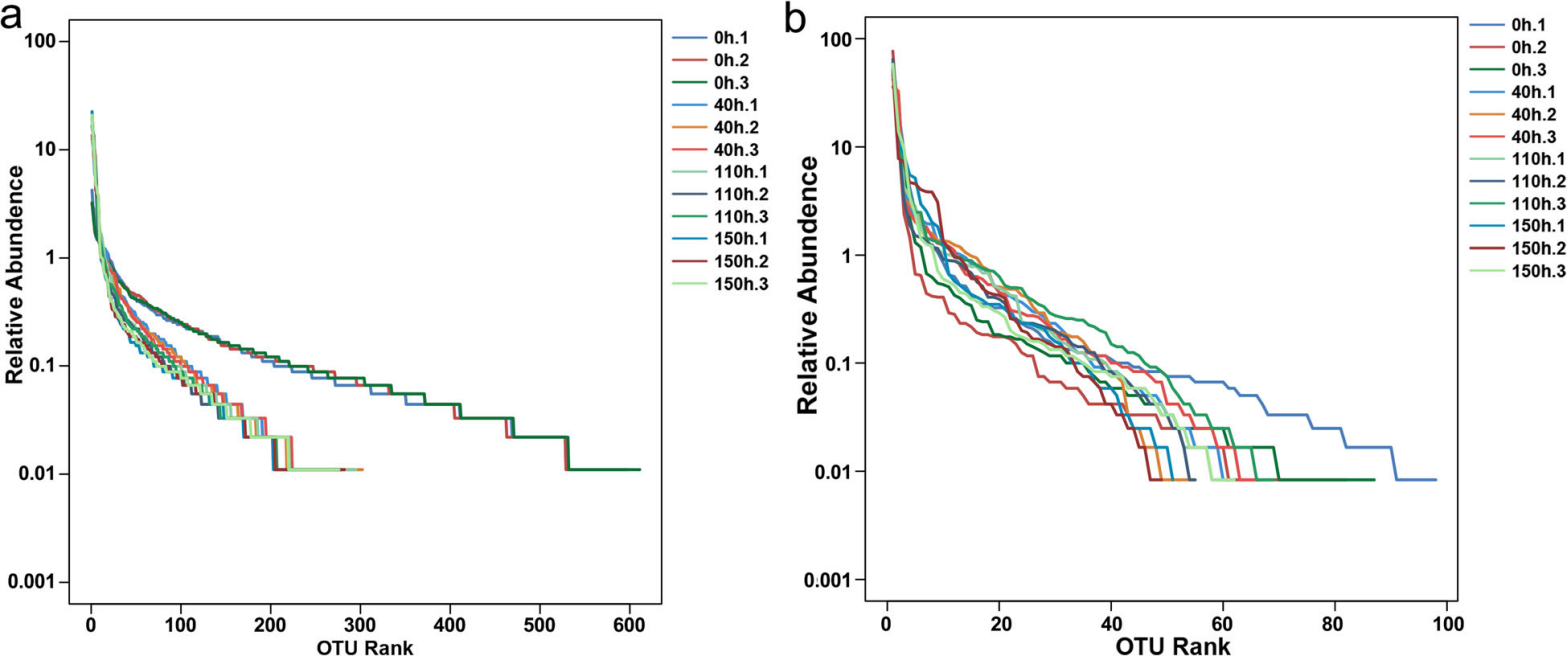
Rank Abundance curves of 12 samples. The different color represent different samples. **a .** Rank Abundance curves of bacteria; **b .** Rank Abundance curves of fungi

We then collected liquid samples at 0, 40, 110 and 150 h post retting and subjected to 16S/18S rRNA sequencing. A total of 167,321 and 181,887 raw reads was generated from 16S and 18S rRNA sequencing data, respectively. After removing the low-quality reads and chimera, the sequence length of trimmed reads is mostly distributed at 420 bp - 490 bp in bacteria, and the fungus sample is mostly distributed at 399 bp - 409 bp. The final rank abundance curve tends to a plateau, indicating that the sample species are richer in composition and higher in uniformity (Fig. 1). The higher species rank value of samples at 0 h (500–600) compared with of samples at 40, 110 and 140 h (200–300) indicated that the fermentation significantly decreased bacterial diversity. In addition, we found the retting significantly reduced the bacterial alpha diversity estimators like Chao 1, PD_whole_tree, Shannon and Simpson index (Table 2). In addition, retting also decreased fungal alpha diversity estimators including Chao 1 and PD_whole_tree, but increased Goods coverage (Table 2). These changes suggested retting decreased microbes viable count and bacterial diversity but increased fungal diversity.

**Table 2:** The alpha diversity of the 16S and 18S rRNA-seq

Group	16S rRNA-seq				
	Chao 1	Goods coverage	PD_whole_tree	Shannon	Simpson
0 h	642.47 ± 8.45 ^a^	0.9920 ± 0.0002 ^a^	25.62 ± 0.21 ^a^	8.01 ± 0.05 ^a^	0.9924 ± 0.0006 ^a^
40 h	388.16 ± 13.70 ^b^	0.9912 ± 0.0003 ^b^	12.83 ± 0.14 ^b^	5.53 ± 0.06 ^b^	0.9447 ± 0.0015 ^b^
110 h	341.90 ± 12.46 ^c^	0.9923 ± 0.0003 ^a^	11.52 ± 0.43 ^c^	5.09 ± 0.17 ^c^	0.9275 ± 0.0102 ^c^
150 h	353.47 ± 5.32 ^c^	0.9919 ± 0.0003 ^a^	12.25 ± 0.19 ^b^	4.75 ± 0.06 ^d^	0.9058 ± 0.0043 ^d^
	**18S rRNA-seq**				
0 h	98.86 ± 4.15 ^a^	0.9988 ± 0.0004 ^b^	3.09 ± 0.30 ^a^	2.26 ± 0.59 ^b^	0.58 ± 0.14 ^b^
40 h	62.75 ± 3.77 ^b^	0.9996 ± 0.0001 ^a^	2.27 ± 0.32 ^bc^	3.01 ± 0.07 ^a^	0.75 ± 0.01 ^a^
110 h	65.79 ± 3.94 ^b^	0.9996 ± 0.0000 ^a^	2.45 ± 0.23 ^b^	2.75 ± 0.30 ^ab^	0.64 ± 0.05 ^ab^
150 h	56.10 ± 8.06 ^b^	0.9997 ± 0.0002 ^a^	1.92 ± 0.07 ^cd^	2.78 ± 0.24 ^ab^	0.69 ± 0.04 ^ab^

Difference in one-way ANOVA is presented by different letters

### Identification of key bacteria responsible for the degumming of kenaf bast

**Fig. 2:**
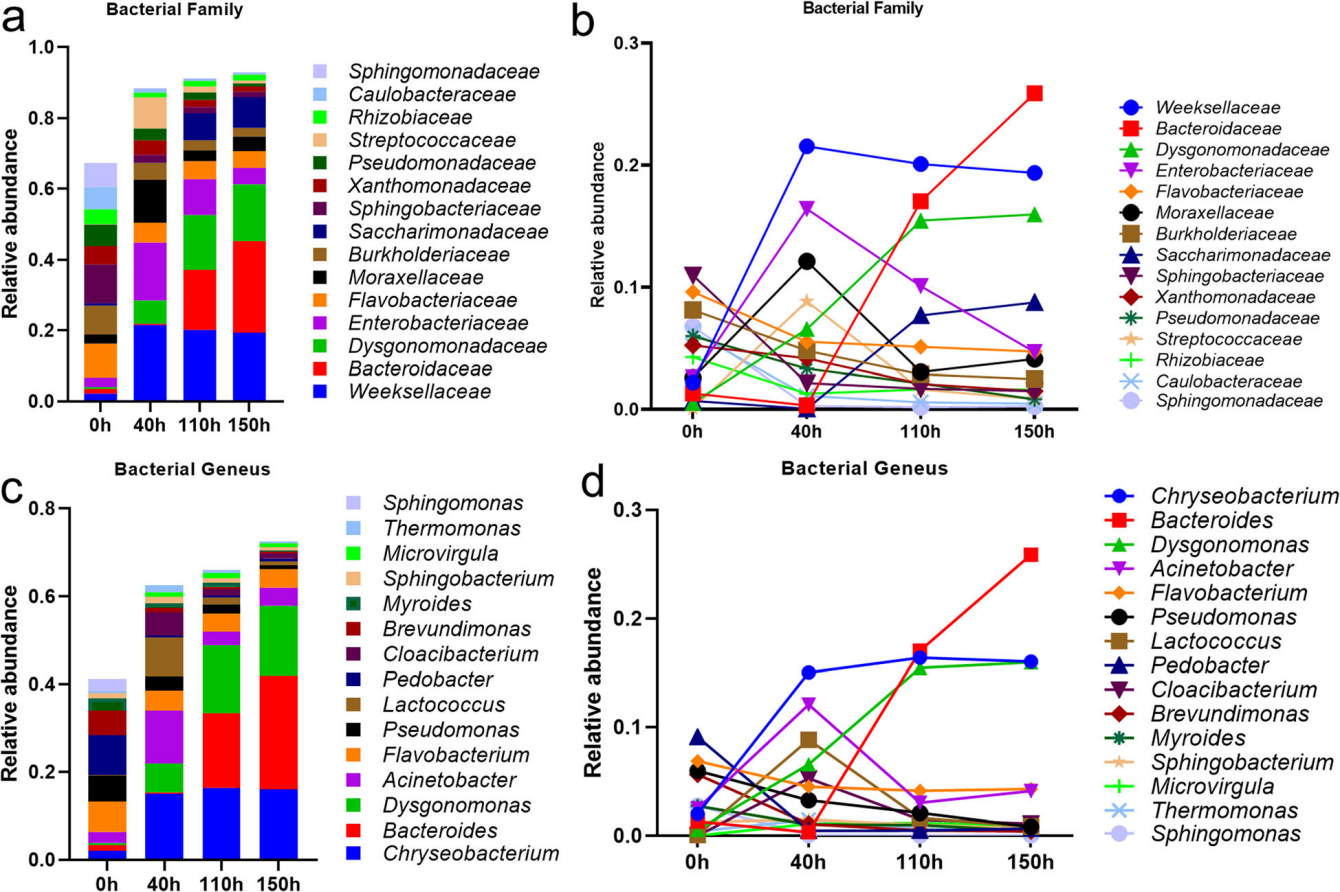
The relative abundance of the dominant bacterial family and genus. **a** and **b**, the stacked and linear figure of the relative abundance of 12 bacterial families (relative abundance > 1%) during the degumming of kenaf bast, respectively. **c** and **d**, the stacked and linear figure of the relative abundance of 9 bacterial genera (relative abundance > 1%) during the degumming of kenaf bast, respectively

After OTUs (operational taxonomic units) annotation, we identified the abundances (at phylum level) of *Bacteroidetes* (from 34.91% at 0 h to 67.75% at 150 h) and *Patescibacteria* (1.00 to 9.53%) were gradually increased during the degumming of kenaf bast (Additional file 1: Figure S1), which replaced the *Proteobacteria*. The initial abundance of *Firmicutes* (2.83%) was firstly increased to 15.28% at 40 h and then decreased to 3.40% at 150 h **(**Additional file 1: Figure S1a and b). At the family level, *Sphingobacteriaceae* (10.98%, *Bacteroidetes*), *Flavobacteriaceae* (9.62%), *Burkholderiaceae* (8.13%), and *Sphingomonadaceae* (6.77%) were the dominant bacteria at the initial (Fig. 2a and b). However, they were replaced by the fast-growing [Weeksellaceae] (21.55%, *Bacteroidetes*), *Enterobacteriaceae* (16.41%, *Proteobacteria*) and *Moraxellaceae* (12.13%, *Proteobacteria*) families at 40 h post retting. The latter bacteria were gradually replaced by the *Bacteroidaceae* family from 40 h to 150 h (25.89%; Fig. 2a and b). We also identified that the growth of *Cytophagaceae* and *Chitinophagaceae* families (*Bacteroidetes*) were inhibited by retting process. Similar changes were found in several bacterial genera. Dominant genera, including *Pedobacter* (9.10%), *Flavobacterium* (6.86%), *Pseudomonas* (5.97%) and *Brevundimonas* (5.64%) kept an equivalent level at the initial (0 h). *Chryseobacterium* (15.03%, [Weeksellaceae]), *Acinetobacter* (12.10%, *Moraxellaceae*) and *Lactococcus* (8.84%, *Streptococcaceae* family) grew to be the dominant bacteria at 40 h, which were then replaced by *Bacteroides* (25.89%) in the fermentation liquid, followed by *Chryseobacterium* (16.03%) and *Dysgonomonas* families (15.96%) (Fig. 2c and d). These changes in bacterial abundances were in response to that of the bacterial viable count in Table 1. These data showed that *Acinetobacter*, *Chryseobacterium*, *Lactococcus* and *Bacteroidetes* at genus level and [Weeksellaceae], *Enterobacteriaceae, Moraxellaceae* and *Bacteroidaceae* at family level might be key candidate bacteria responsible for the degumming of kenaf bast.

### Identification of key fungi responsible for the degumming of kenaf bast

**Fig. 3:**
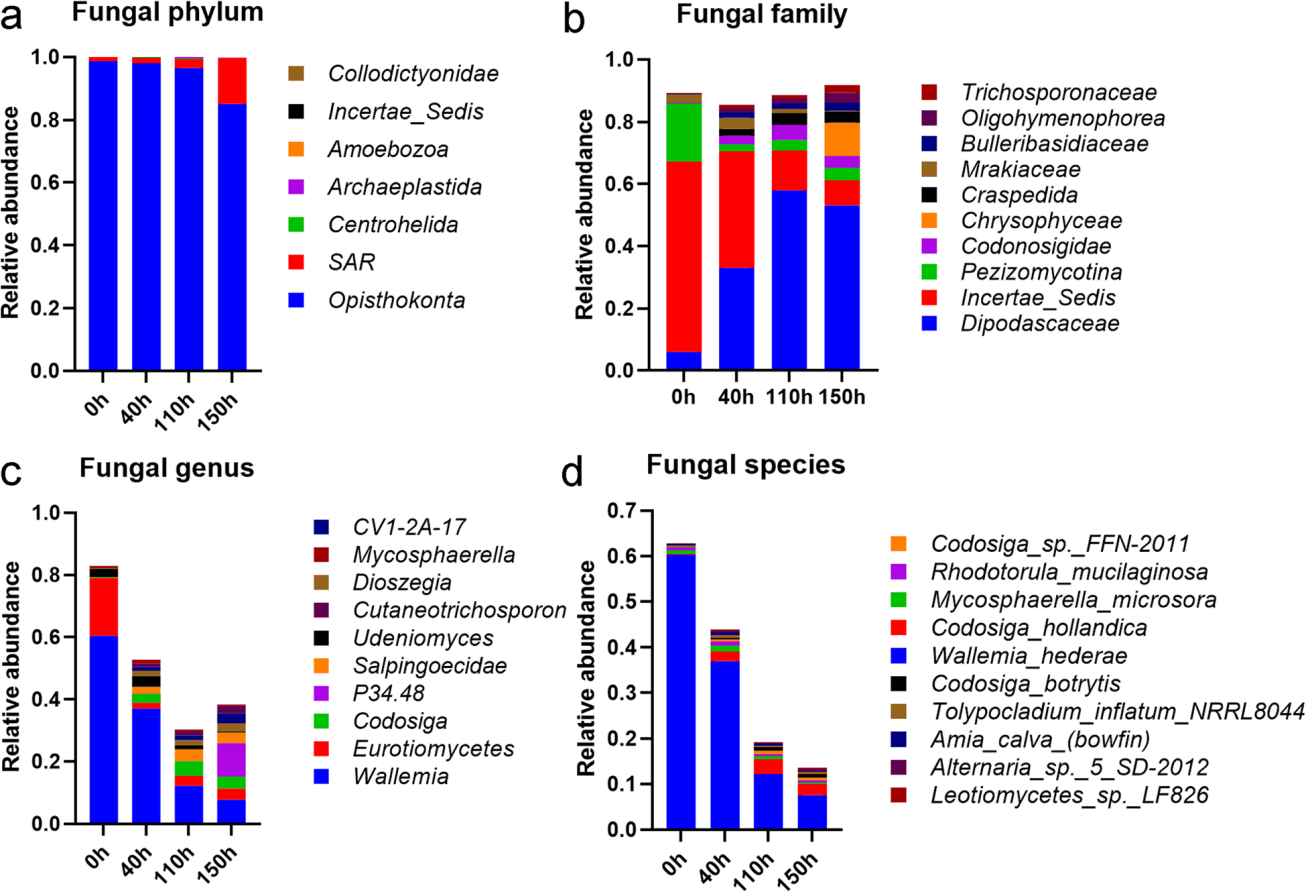
The relative abundance of the dominant fungal family. **a** to **d**, the stacked figure of the relative abundance of dominate fungal at phylum, family, genus and species level during the degumming of kenaf bast, respectively

As expected, fungal abundances were also changed in response to degumming. All fungi were mainly dominanted by 2 phyla: *Opisthokonta* (98.73%) and *SAR* (1.24%). The relative abundance of *Opisthokonta* subkingdom was gradually decreased to 85.09%, and replaced by *SAR* phylum (14.61% at 150 h; Fig. 3a). The dominant fungal families *Incertae Sedis* (61.11 to 8.10%) and *Pezizomycotina* (18.73 to 3.89%) were replaced by *Dipodascaceae* (5.98 to 53.08%) and some other fungi such as *Bulleribasidiaceae, Craspedida, Chrysophyceae*, etc. (Fig. 3b). At genus level, the results showed that *Wallemia* (60.43%) and *Eurotiomycetes* (18.55%) were the dominant fungi (Fig. 3c). As for specific species, the dominant positions of *Wallemia hederae* (60.33%) at the initial, but decreased at 40 h (36.97%), 110 h (12.12%) and 150 h (7.50%) (Fig. 3d). The relative abundance of *Codosiga hollandica* species was increased from 0.16% at 0 h to 2.42% at 150 h.

### Microbia secretomics analysis and identification of candidate proteins or peptides

**Fig. 4:**
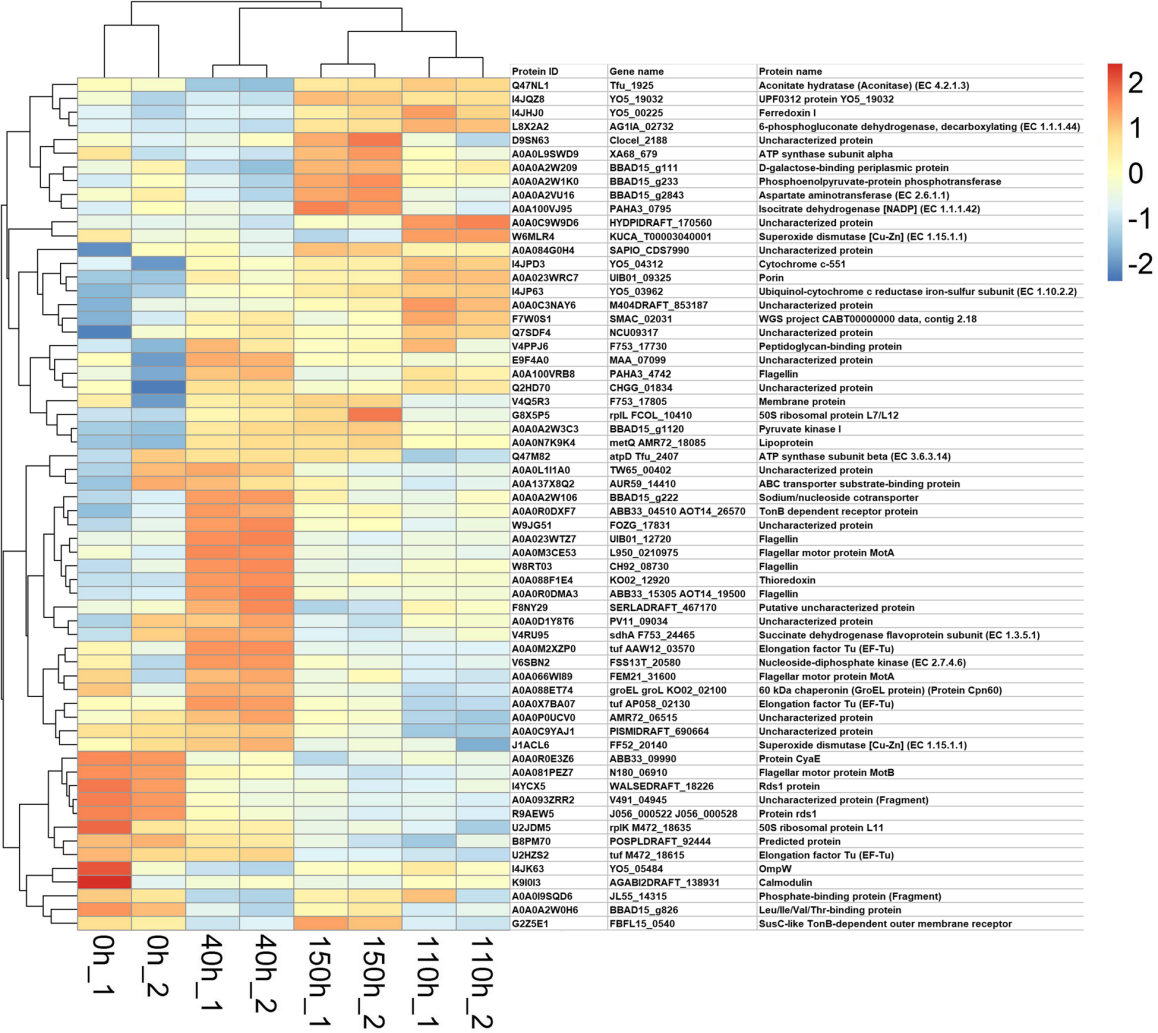
The heatmap of the 64 differentially expressed proteins/peptides. Red and blue represents the high and low expression, respectively. _1 and 2 represent biological repeat 1 and 2 in each group, respectively

We then performed the secretomics analysis to identify the candidate proteins which might be responsible for biological degumming of kenaf bast, since there are significant changes in the relative abundance of bacteria and fungi. A total of 197 proteins, including 67 DEPs were identified (Additional file 2: Table S1). Clustering analysis showed the distinct expression patterns of these proteins in the samples (Fig. 4). We identified the significantly down regulated Rds1 protein peptides (including I4YCX5 and R9AEW5, A1DDU4, A0A0S7E3J2, A0A0J5SQP1, Q4WVL1, B0Y1F6, A0A084BN00 and A0A0K8L4F0), superoxide dismutase peptides (J1ACL6 and A0A0Q9DZS2), and the upregulated peptides of pyruvate kinase I (A0A0A2W3C3), lipoprotein (A0A0N7K9K4), ferredoxin I (I4JHJ0), thioredoxin (A0A088F1E4, A0A0M2Y158 and A0A0M3C9P8). A0A0A2W3C3 was enriched into the pathways including glucagon signaling pathway (ko04922) and pyruvate metabolism (ko0492). A peptide of aconitatehydratase (aconitase, ACO), which is related to the glyoxylate and dicarboxylate metabolism (ko00630), was decreased at 40 h and then increased at 110 and 150 h post retting compared with 0 h (Table 3). Most of the other peptides were annotated with transporter activities (Additional file 2: Table S1).

**Table 3:** Several differentially expressed proteins in during degumming

Protein ID		Time (s)			Protein name	Gene ontology	Pathway
	0 h	40 h	110 h	150 h			
R9AEW5;A1DDU4;A0A0S7E3J2; A0A0J5SQP1;Q4WVL1;B0Y1F6; A0A084BN00;A0A0K8L4F0	4391.5	715.0	340.8	362.7	Protein rds1		
A0A0N7K9K4	889.5	2642.3	1897.0	2776.1	Lipoprotein		
A0A023WRC7	308.7	614.1	981.2	671.3	Porin	integral component of membrane [GO:0016021]; porin activity [GO:0015288]	
I4YCX5	15,628.3	386.7	228.8	254.1	Rds1 protein		
A0A0A2W3C3	964.2	4595.7	2396.7	5424.1	Pyruvate kinase I		ko04922: Glucagon signaling pathway; ko0492: Pyruvate metabolism
I4JHJ0	580.1	651.8	2090.5	1518.2	Ferredoxin I		
Q47NL1	816.8	199.8	2134.1	1615.2	Aconitate hydratase (Aconitase) (EC 4.2.1.3)	4 iron, 4 sulfur cluster binding [GO:0051539]; aconitate hydratase activity [GO:0003994]; metabolic process [GO:0008152]	ko00630: Glyoxylate and dicarboxylate metabolism
A0A088F1E4;A0A0M2Y158; A0A0M3C9P8	201.3	1957.3	429.8	1047.9	Thioredoxin	protein disulfide oxidoreductase activity [GO:0015035]; cell redox homeostasis [GO:0045454]; glycerol ether metabolic process [GO:0006662]	
J1ACL6;A0A0Q9DZS2	393.9	447.6	286.3	325.2	Superoxide dismutase [Cu-Zn] (EC 1.15.1.1)	metal ion binding [GO:0046872]; superoxide dismutase activity [GO:0004784]	
W6MLR4;W0TF05;A0A090C5A2	1289.9	1014.2	2316.6	770.7	Superoxide dismutase [Cu-Zn] (EC 1.15.1.1)	metal ion binding [GO:0046872]; superoxide dismutase activity [GO:0004784]	
V4RU95;S6LCD6;M2UZN0;L0GJW1; I4JML0;I4CTX4;H7F0P7;F8H871; F2MXE8;A4VKP6;A0A137Y7T1; A0A137WWW6;A0A0I9SRB2; A0A0H3YZE1;A0A0D7EA74;A0A0C2MWX1; A0A098FQU9;A0A061JVN2;A0A023WTY8	1525.4	2439.2	1244.5	1037.6	Succinate dehydrogenase flavoprotein subunit (EC 1.3.5.1)	plasma membrane [GO:0005886]; flavin adenine dinucleotide binding [GO:0050660]; succinate dehydrogenase (ubiquinone) activity [GO:0008177]; electron transport chain [GO:0022900]; tricarboxylic acid cycle [GO:0006099]	
A0A0D1Y8T6	1129.9	1915.8	958.2	658.1	Uncharacterized protein	succinate dehydrogenase activity [GO:0000104]	
H7FVF4	147.3	38.6	124.9	289.2	Isocitrate lyase (EC 4.1.3.1)	isocitrate lyase activity [GO:0004451]; carboxylic acid metabolic process [GO:0019752]	

Among the other non-DEPs, we identified that the peptide of Aldehyde dehydrogenase family protein (A0A160F3I4), Aspartate aminotransferase (A0A0A2VU16) and 6-phosphogluconate dehydrogenase (L8X2A2). The L8X2A2 was identified to be related with pentose phosphate pathway.

## Discussion

The degumming of kenaf bast is a process mediated by dynamic change of microbes. Using the 16S/18S rRNA sequencing, we identified the changed bacterial and fungal abundance during the degumming of kenaf bast (0~ 150 h). In the fermentation liquid, the growth of *Cytophagaceae* and *Chitinophagaceae* was inhibited during the degumming of kenaf bast. Many bacteria genera played crucial roles in in the degumming process of kenaf bast, such as *Bacteroides*, *Chryseobacterium*, *Dysgonomonas, Acinetobacter,* and *Lactococcus*, of which the abundance were greatly changed with degumming treatment. Similarly, some fungi also participated in the degumming process of kenaf bast including *Pezizomycotina, Dipodascaceae, Codosiga hollandica,* and *Incertae Sedis*. The abundance of subdivided *Wallemia* and *Eurotiomycetes* genera were dramatically reduced in the process of dealkylation and fermentation. And the increased *Dipodascaceae* family might promote the degumming of kenaf bast. A series of *Bacillus* strains has been identified to be ramie- or kanef-degumming strains, like *B. cereus* hn1–1 [10], *B. pumilus* [7], *B. licheniformis* and *B. subtilis* [11] and *B. tequilensis* SV11-UV37 [6]. In addition, our previous study [7] showed that seven bacterial strains belonging to the species including *B. pumilus, B. alcalophilus, C. tertium, Brevibacillus brevis, Pectobacterium carotovora, Erwinia chrysanthemi* and *Tyromyces* sub *caesius* were the key in strains for the degumming of kenaf bast. Other reports also showed the ability of *B. licheniformis*, *Paenibacillus macerans*, *C. tertium, B. tequilensis* and *B. vulgatus*or the proteases and pectinolytic enzymes derived from these strains for degumming fiber, wool and wood [6, 7, 13, 14]. For instance, enzymatic treatment is an acceptable method of intervention among the methods for wool treatment for breaking down the surface structure [14]. Serine proteases are the most common commercial proteases derived from *Bacillus* strains.

For the degumming of plant fibers, some researchers had isolated proteases, xylanases and pectate lyases from the bacteria like *Acinetobacter spp. (> 1 species of the genus)* [15] and *B. cereus* [16] and fungi including Extremophilic fungi [17–19]. Researchers also identified the lignin degrading role of *Pseudomonas*, *Lactococcus* and *Acinetobacter* strains in hemp, ramie and mechanical pulp [20–23]. For instance, Hu et al. [23] observed that abundances of *Pseudomonas* and *Acinetobacter* were increased to the highest at 36 h post retting and decreased subsequently. In particular, the finding about *Acinetobacter* and *Lactococcus* was consistent with our results, which was increased to 12.09 and8.84% at 40 h and then decreased to 4.10 and 0.84% at 150 h. The dynamic changes of these bacteria during the degumming of kanef bast suggested their crucial roles in degrading kanef.

Kanef-degumming is a dynamic process of bacterial adaptation and growth. The initial stage is characterized by decreased bacterial richness and diversity [24]. We determined the decreased bacterial viable count at the 40 h post retting, followed by increased bacterial viable count but not bacterial richness and diversity. Our present study presented a cluster of anaerobic *Bacteroidaceae* members like *Bacteroides*, *Chryseobacterium* and *Dysgonomonas*, played crucial roles in the degumming of kanef bast, especially in the late stage. *Cytophagaceae* was initially inhibited, which might guarantee the fiber structure. The rapid growth of anaerobic *Bacteroidaceae* bacteria changed bacterial diversity. Xylan and pentose (including xylose) are main components of hemicellulose in plants [25]. The degradation of hemicellulose into oligomers and sugarsis a metabolic property shared by sugar-fermenting *Bacteroides* [26–29]. The increased abundance of these *Bacteroidaceae* members might suggest the accumulation of their substrates derived from the early stage fermentation from aerobic bacteria like *Acinetobacter* and *Lactococcus* or the changed environments.

In addition, we also identified the down regulation of several peptides of Rds1 during the degumming of kanef bast. Rds1 a stress-responsible protein, which could be depressed by starving from glucose, ammonium, phosphate, exposuring to carbon dioxide and high temperature [30]. The down regulation of it was theoretically in line with the hypothesis that the starvation of sugar and oxygen of early retting stage. What’s more, the identification of the gradually decreased halophilic *Wallemia hederae* and increased turfgrass pathogen in the fermentation liquid might suggest the deterioration of fermentation. *Codosiga hollandica.*

## Conclusions

In conclusion, we identified a cluster of key bacteria responsible for the degumming of kanef bast. We identified that the growth of *Cytophagaceae* was initially inhibited at the early stage of degumming for kenaf bast. The up-and-down change in the abundance of *Acinetobacter* and *Lactococcus* (*Streptococcaceae*) and the gradually increased growth of *Bacteroides*, *Chryseobacterium*, *Dysgonomonas* characterized the degumming process. In addition, we also identified the increased *Codosiga hollandica* and decreased *Wallemia hederae* fungus family during degumming for 150 h. Secretory proteomics analysis showed Rds1, pyruvate kinase I and aconitatehydratase peptides were changed during the degumming of kanef bast. These findings provide evidence on the crucial roles of these microbes in the degumming of kenaf bast.

## Methods

### Bacteria collection and degumming of kenaf bast

Humus samples (50 g) were collected from Sanya, China. Water samples (100 ml) were collected from a conventional retting pond (50 cm away from the water surface) in Xiaoshan, Zhejiang, China. Soil samples (50 g) were collected from continuous cropping soil of Kenaf in Xiaoshan. Soil and humus samples were diluted into 100 ml bacteria free water (autoclave at 121 °C for 20 min), filtered and then mixed with the above water samples.

Kenaf bast was collected from Xianghongma No. 1 plants in Changsha, China. The samples were cut into pieces (3 cm) and then immersed into bacteria mixture (10 g: 5 ml) with supplementation of 100 ml bacteria free water. For the degumming of kenaf bast, samples were maintained on an orbital shaker at 30 °C, pH 7.0, for 30 min. Then the fermentation liquid samples were collected at 0, 40, 110, 150 and 190 h post degumming and used for further analysis. Each experiment was done in triplicates.

### Determination of kenaf bast weight loss rate and viable count of bacteria

The weight loss rate of the kenaf bast samples in each condition was calculated according to the following formula: weight loss rate (%) = [initial weight (10 g)-final weight (g) of kenaf bast]/initial weight (10 g) of kenaf bast × 100%. Total viable count was quantified traditionally using the colony-forming units (CFUs) after incubation on nutrient broth solid media (pH 7.0) for 0–190 h.

### DNA extraction and 16S and 18S ribosomal RNA gene sequencing

DNA extraction was performed using a PowerSoil™ DNA Isolation Kit (MOBIO laboratories, San Diego, Carlsbad, California, USA). The concentration and purity of the DNA was measured by agarose gel electrophoresis. The 16S ribosomal DNA (rDNA) gene V3-V4 region of the bacteria was amplified by PCR with bar coded primers (343F: 5′- TACGGRAGGCAGCAG − 3′ and 798R: 5′- AGGGTATCTAATCCT-3′), using FastPfu Polymerase (TransStart, Beijing, China). The PCR primers for the 18S rDNA of fungus were NS1: 5′- GTAGTCATATGCTTGTCTC − 3′ and NS8: 5′- TCCGCAGGTTCACCTACGGA − 3′. Reaction parameters were: 95 °C for 5 min, followed by 30 cycles of 95 °C 30 s, 52 °C 45 s (16S) or 1 min (18S), 72 °C 1 min, and the final step of 72 °C for 10 min. The amplicons of 16S and 18S rDNA were purified by an AxyPrep DNA Gel Extraction Kit (Axygen Biosciences, Union City, California, USA). After repeating the above steps (amplification and purification), the concentration of final purified amplicons was detected by Qubit 2.0 (Thermo Fisher Scientific, Walthan, Massachusetts, USA). At the end, the samples were pooled and subjected to an Illumina MiSeq Instrument (Illumina, San Diego, California, USA) in Shanghai OE Biotech. Co., Ltd. with 350 bp paired-end sequencing.

### Data processing

Raw data from different samples were identified based on the unique barcode. The primer sequences were removed and data were trimmed using U-Search software [31]. FLASH v1.2.7 software (http://ccb.jhu.edu/software/FLASH) was used for merging paired-end reads and the counting of reads [32]. The chimeric sequences were removed using UCHIME (http://www.drive5.com/usearch/index.html) [33]. Sequences were clustered into OTUs by QIIME (v1.8.0, http://qiime.org/) [34] according to the minimal 97% similarity. Through matching to the Silva database (https://www.arb-silva.de/) [35], the taxonomic information for each OTU was obtained. Alpha and beta diversities were analyzed to determine differences among groups in terms of species complexity by QIIME (v1.8.0) software.

### Protein extraction, digestion, and iTRAQ labeling

Fermentation liquid samples (30 ml) were collected at 0 h, 40 h, 110 h and 150 h, and then centrifuged at 1500 g for 10 min in an Eppendorf centrifuge (Eppendorf, San Diego, California, USA). The supernatants were collected and filtered through a 0.22 μm membrane. Samples were then diluted into precooled TCA/acetone (1:9) solutions (1: 4 v/v) and then stored at − 20 °C overnight. Pellets were collected by centrifugation (Sigma Aldrich, Schnelldorf, Germany) at 17000 g for 30 min, followed by washing with precooled acetone (90%) for three times. The precipitate was air-dried and then dissolved in sodium dodecyl sulfonate lysate supplementing (Beyotime, Shanghai, China) with protease inhibitor cocktail (P8340, Sigma, USA) on a homogenizer (Hai Shu Ke Sheng, Ningbo, Zhejiang, China). The crude precipitates were collected by centrifugation at 12000 g for 10 min at 4 °C (Sigma Aldrich, Schnelldorf, Germany). The supernatant was selected after sonication by centrifugation (12,000 g for 15 min) for twice. Finally, the supernatant was stored at − 80 °C for further use. The concentration of protein was measured using BCA method [36], with BCA Protein Assay Kit (Thermo Scientific Dionex, San Jose, USA). The integrity of the extracted protein was detected by SDS-PAGE [37].

The quantified samples were then digested according to the filter aided sample preparation procedure as previously described [38]. In brief, 100 μg of protein was precipitated by precooled acetone (1:5 v/v) at − 20 °C for 1 h, centrifuged at 16000 g for 10 min at 4 °C, and vacuum freeze-dried. Protein precipitation was prepared using an iTRAQ kit (Applied Biosystems, Carlsbad, California, USA) following the manufacturer’s instructions. The marked samples were then mixed, dried and then subjected to separation and identification.

### 2D-LC-MS/MS analysis

The freeze-dried sample was dissolved in 110 μL of the mobile phase A solution. Peptide separation was performed on an Agilent 1200 HPLC (Agilent Technologies, Foster City, California, USA) with the Narrow-Bore column (2.1 mm × 150 mm × 5 μm), analytical guard column (4.6 × 12.5 mm, 5-Micron), flow rate of 0.3 ml/min, at 210 nm and 280 nm in Shanghai Luming Biotech. Co., Ltd. Reverse phase chromatographic analyses were performed using Nano-RPLC Buffer A (Applied Biosystems), PepMap100C18 column (75 μm × 20 mm, 3 μm, NanoViper; Thermo Scientific Dionex, San Jose, USA) with the mobile phase B increased from 5 to 35% in 70 min. The Q Exactive Orbitrap mass spectrometer (Thermo Fisher Scientific, Bremen, Germany; nano-electrospray ionization, 1.6 kV, 250 °C) was used for data-dependent acquisition according to the previously reported method [39].

### Protein identification and quantification

The raw proteomics data in the format of .raw was aligned to UniProt database (https://www.uniprot.org/) using Maxquant 1.5.1.0 (Version 1.5.1.0; Thermo Fisher Scientific). Proteins and peptides with fold discovery rate < 0.01 were retained as for further identification of differentially expressed peptides/proteins (DEPs). The significant different proteins between groups were identified with the threshold of T-test *p* value ≤0.05 and fold change (FC) ≥ 1.2.

### Bioinformatics analysis

For annotation of the DEPs, Gene Ontology (GO, http://www.geneontology.org) and Kyoto Encyclopedia of Genes and Genomes (KEGG, http://www.genome.jp/kegg/) databases were used for the gene functions prediction. The GO classifications of molecular function, biological process and cellular component and the pathways significantly related to these DEPs were identified with the criteria of *p* < 0.05.

### Statistical analysis

Data were expressed as the mean ± standard deviation. The SPSS 22.0 software was employed for the statistical analysis. One-way ANOVA test was performed to analyze the differences. Comparison of differences between groups was detected using t-test. The *p*-value < 0.05 was considered as significantly difference.

## Supplementary information

**Additional file 1 Figure S1.** The relative abundance of the dominant bacterial phyla. a and b, the stacked and linear figure of the relative abundance of 8 phyla during the degumming of kenaf bast, respectively.

**Additional file 2 Table S1.** The list of the differentially expressed proteins/peptides in the degumming of the kenaf bast.

## Data Availability

The original data were uploaded to SRA database (https://www.ncbi.nlm.nih.gov/sra) and the BioProject ID is PRJNA562024.

## Abbreviations

ACO: Aconitase
CFUs: Colony-forming units
DEPs: Differentially expressed proteins/peptides
FC: Fold change
GO: Gene Ontology
iTRAQ: Isobaric tag for relative and absolute quantitation
KEGG: Kyoto Encyclopedia of Genes and Genomes
OTUs: Operational taxonomic units (OTUs)
rDNA: Ribosomal DNA
