# Supplementary figures and images for: Insights on bio-degumming of kenaf bast based on metagenomic and proteomics
Source: BMC Genomics. 2020 Feb 3;21:121. doi: 10.1186/s12864-020-6531-2 (PMC6998070; doi:10.1186/s12864-020-6531-2)

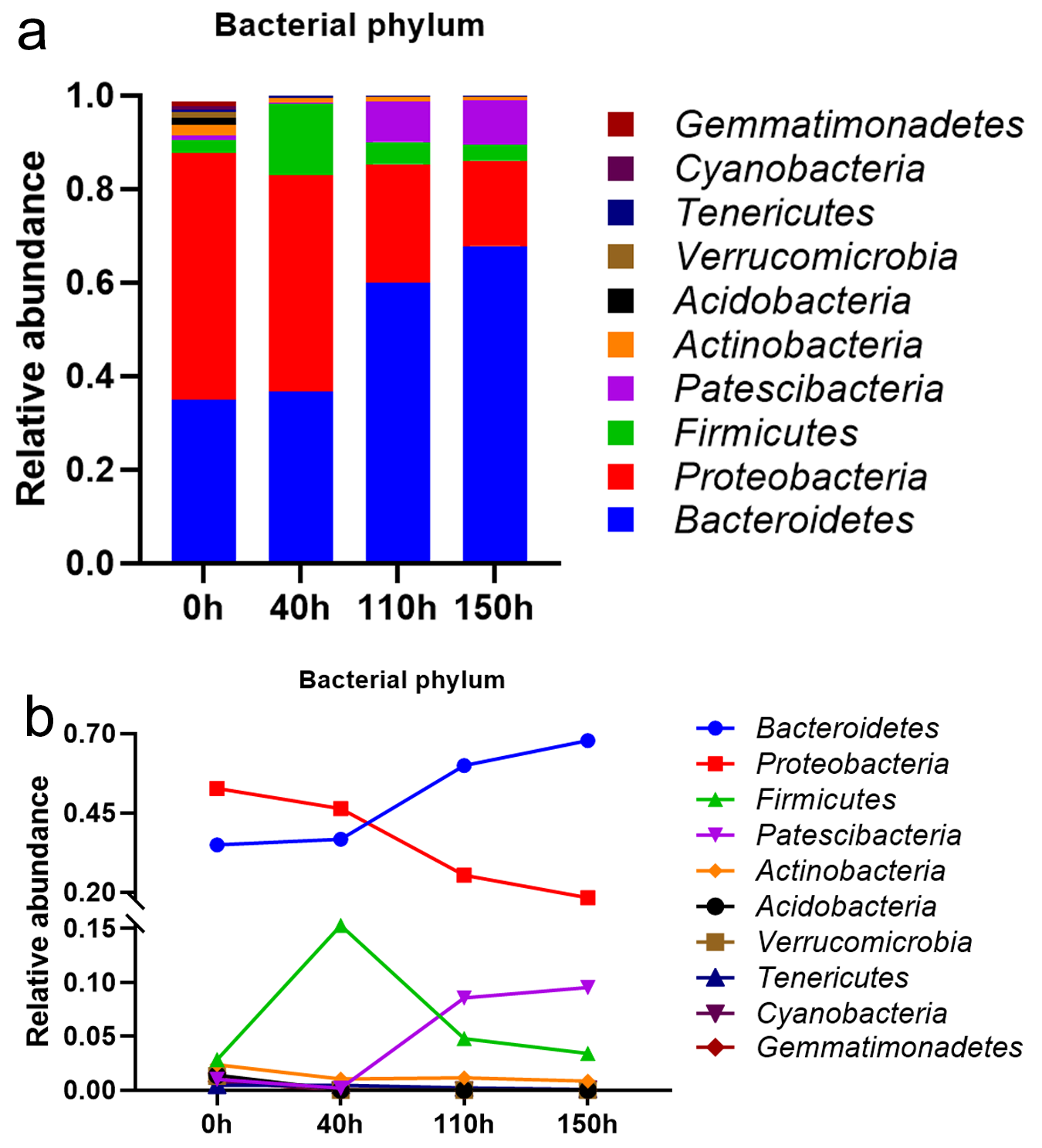

Supplement: Supplementary file 1 — Additional file 1 Figure S1. The relative abundance of the dominant bacterial phyla. a and b, the stacked and linear figure of the relative abundance of 8 phyla during the degumming of kenaf bast, respectively. [file 12864_2020_6531_MOESM1_ESM.tif]
